# Single isocenter stereotactic radiosurgery for patients with multiple brain metastases: dosimetric comparison of VMAT and a dedicated DCAT planning tool

**DOI:** 10.1186/s13014-019-1315-z

**Published:** 2019-06-11

**Authors:** Jan Hofmaier, Raphael Bodensohn, Sylvia Garny, Indrawati Hadi, Daniel F. Fleischmann, Michael Eder, Yavuz Dinc, Michael Reiner, Stefanie Corradini, Katia Parodi, Claus Belka, Maximilian Niyazi

**Affiliations:** 1 Department of Radiation Oncology, University Hospital, LMU Munich, Marchioninistraße 15, 81377 Munich, DE Germany; 2 German Cancer Consortium (DKTK), Munich, Germany; 3 German Cancer Research Center (DKFZ), Heidelberg, Germany; 4 Department of Medical Physics, Faculty of Physics, LMU Munich, Munich, Germany

**Keywords:** Stereotactic radiosurgery, Brain metastases, Dynamic conformal arc therapy, Single Isocenter, VMAT

## Abstract

**Background:**

In this dosimetric study, a dedicated planning tool for single isocenter stereotactic radiosurgery for multiple brain metastases using dynamic conformal arc therapy (DCAT) was compared to standard volumetric modulated arc therapy (VMAT).

**Methods:**

Twenty patients with a total of 66 lesions who were treated with the DCAT tool were included in this study. Single fraction doses of 15–20 Gy were prescribed to each lesion. Patients were re-planned using non-coplanar VMAT. Number of monitor units as well as V_4Gy_, V_5Gy_ and V_8Gy_ were extracted for every plan. Using a density-based clustering algorithm, V_10Gy_ and V_12Gy_ and the volume receiving half of the prescribed dose were extracted for every lesion. Gradient indices and conformity indices were calculated. The correlation of the target sphericity, a measure of how closely the shape of the target PTV resembles a sphere, to the difference in V_10Gy_ and V_12Gy_ between the two techniques was assessed using Spearman’s correlation coefficient.

**Results:**

The automated DCAT planning tool performed significantly better in terms of all investigated metrics (*p* < 0.05), in particular healthy brain sparing (V_10Gy_: median 3.2 cm^3^ vs. 4.9 cm^3^), gradient indices (median 5.99 vs. 7.17) and number of monitor units (median 4569 vs. 5840 MU). Differences in conformity indices were minimal (median 0.75 vs. 0.73) but still significant (*p* < 0.05). A moderate correlation between PTV sphericity and the difference of V_10Gy_ and V_12Gy_ between the two techniques was found (Spearman’s rho = 0.27 and 0.30 for V_10Gy_ and V_12Gy_, respectively, *p* < 0.05).

**Conclusions:**

The dedicated DCAT planning tool performed better than VMAT in terms of healthy brain sparing and treatment efficiency, in particular for nearly spherical lesions. In contrast, VMAT can be superior in cases with irregularly shaped lesions.

## Background

Over the last decades, whole brain radiotherapy (WBRT) has been the only option for patients with multiple cerebral metastases, whereas patients with a limited number and size of the metastases have been treated with stereotactic radiosurgery (SRS). Guidelines suggested a stereotactic approach for up to 4 metastases [1, 2]. Furthermore, due to increased adverse side effects in the ablative treatment of large volume metastases, the size for single metastases was usually limited to a maximum diameter of 3.5 cm, and for multiple metastases to 2.5 cm [1].

In 2014, Yamamoto et al. published a 2014 multicentric prospective trial of 1194 patients treated with Gamma-Knife SRS. The study compared outcome in patients undergoing multiple SRS treatments for 5–10 brain metastases, as compared to patients with 2–4 metastases. Altogether, there was no difference in overall survival or radiation induced adverse effects. The authors concluded that SRS for 5–10 brain metastases was non-inferior to SRS for 2–4 metastases [3]. Furthermore, Yamamoto et al. also evaluated the outcome in patients with more than 10 brain metastases, showing comparable, non-inferior results [4]. The results were confirmed on long-term follow-up in 2017 [5]. With new dedicated techniques, the treatment time for multiple metastases is reduced, making SRS a valid alternative to WBRT for the treatment of multiple brain metastases [6]. These techniques also have the potential to improve the cost-effectiveness of radiosurgery for brain metastases [7].

From a technical perspective, the treatment of multiple metastases with SRS poses a number of challenges. In linear accelerator (LINAC) based SRS for single brain metastases, the dynamic conformal arc therapy (DCAT) technique can be used. This technique enables a plan quality on LINACs which is comparable to robotic techniques [8]. Furthermore, this approach can also be used for multiple metastases, however, it requires an individual isocenter for every lesion and therefore repeated image guidance, resulting in very time consuming treatment delivery. One possibility to achieve the simultaneous treatment of multiple lesions with a single isocenter is the use of intensity modulated techniques, such as RapidArc™ (Varian, Palo Alto, California, USA) or volumetric modulated arc therapy (VMAT) [9], possibly combined with dedicated, automated planning algorithms [6, 10, 11]. Compared to DCAT, intensity-modulated techniques entail a higher plan complexity and a higher number of monitor units [12]. Therefore, DCAT techniques with a reduced number of isocenters [13] or even a single isocenter [14] have been proposed. One such approach is implemented in the Elements™ Multiple Brain Mets SRS software by Brainlab (Munich, Germany): this software extends the DCAT technique to the treatment of multiple targets with a single isocenter. The Department of Radiation Oncology of the University Hospital, LMU Munich is one of the first centers to use this software in combination with Elekta LINACs in clinical practice.

In this dosimetric study, Elements™ Multiple Brain Mets SRS v1.5 (MBSRS) single isocenter DCAT plans were compared to Monaco® (Elekta, Stockholm, Sweden) VMAT plans in terms of plan quality metrics, such as conformity indices, healthy brain sparing and monitor unit (MU) efficiency.

## Methods

### Contouring

For planning purposes, a T1 weighted gradient-echo magnetic resonance imaging (MRI) sequence (magnetization-prepared rapid gradient-echo, MPRAGE) with contrast media and an axial slice thickness of 1 mm was acquired. This sequence was co-registered to the planning computed tomography (CT) image. The time span between MRI and planning CT was as short as possible, but not longer than 1 week. The treatment was performed as soon as possible but not later than 10 days after the MRI. If not contraindicated, contrast media was applied for the planning CT in order to enhance co-registration. Co-registration was performed automatically by the MBSRS tool and was reviewed and, if necessary, adjusted by the treating radiation oncologist. Organs at risk (OAR) such as the brainstem, optical lenses, eyeballs, optical nerves, optical tract, chiasm and hippocampi were automatically contoured by the software and reviewed by a radiation oncologist. The metastases themselves were contoured with consideration of the axial, sagittal and coronal slices. For the planning target volumes (PTV) a 1 mm margin was added. Finally, the contours were reviewed by a senior radiation oncologist.

### Prescription

All metastases were treated with single fraction doses ranging from 15 to 20 Gy. Main criterion for dose prescription was the size of the metastases following current guidelines [1, 2]. In addition the dose prescription was adjusted in order to comply with the dose constraints for OARs. A reduced prescription dose was also used in case lesions were in close proximity to each other. If lesions were directly next to each other, a joint structure was created and treated as one PTV. Doses were prescribed to the 80% isodose level and had to enclose 98% of the target volume. In clinical practice, isodose level values were in the range of 75 to 85%. The dose prescription was determined by a senior radiation oncologist while reviewing the contours and, if necessary, adjusted during treatment planning.

### DCAT planning

**Fig. 1:**
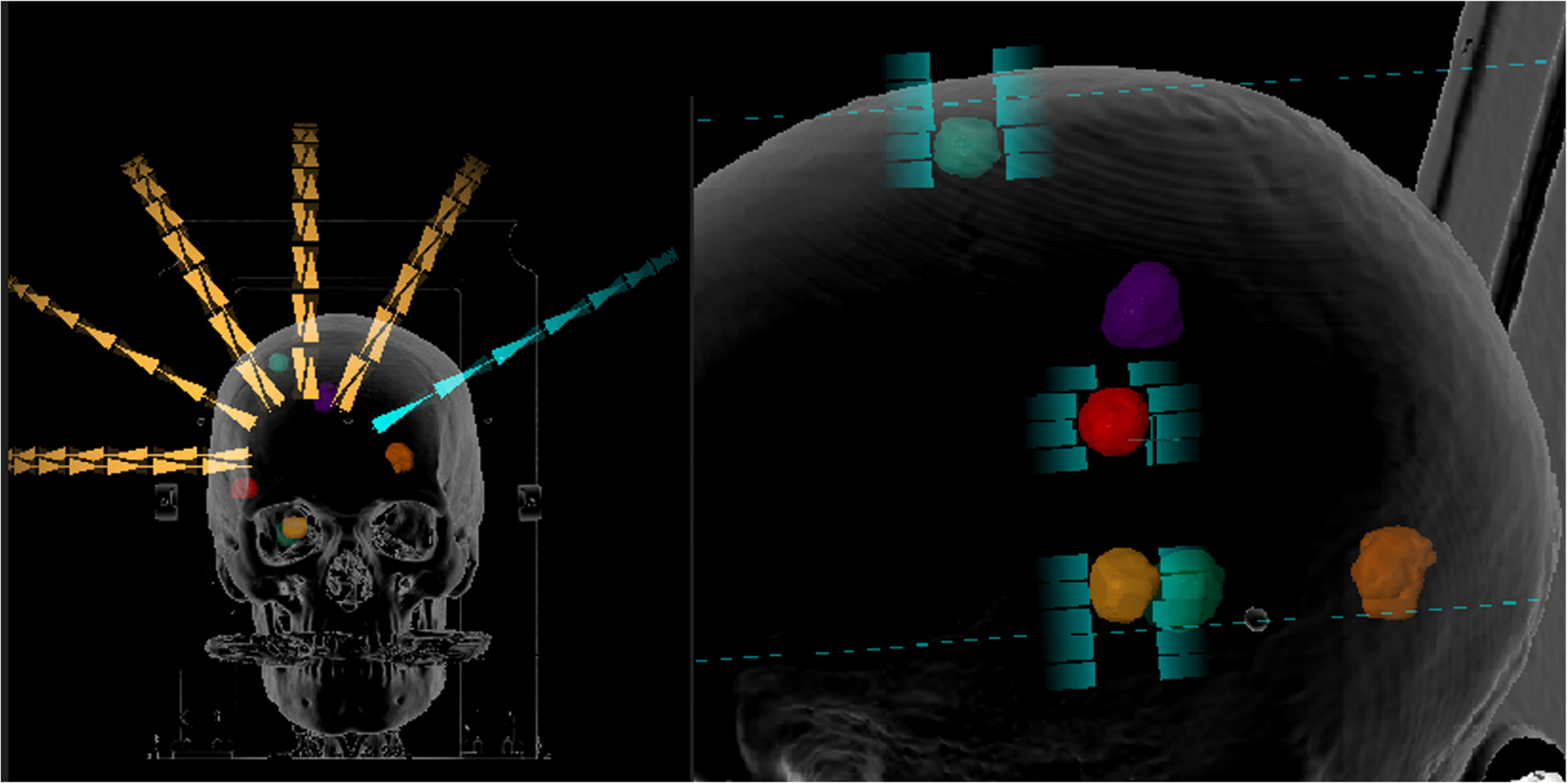
Arc setup and beams-eye-view for one of the arcs for a patient with 6 lesions. 3 of the lesions are treated simultaneously by the selected arc (blue)

The MBSRS tool offers a highly automated planning workflow for single isocenter DCAT treatments of multiple brain metastases. The user chooses a pre-defined template containing the number of couch angles and the minimum collimator angle. In this study, either 5 or 6 couch angles were used, which was decided on an individual basis during treatment planning. The treatment isocenter is set to the geometrical average of the centers-of-mass of all target volumes automatically by the MBSRS tool. At each couch angle, up to two independent arcs in clockwise and counter-clockwise directions are used. The algorithm automatically determines which targets are going to be treated conformally through each arc. To avoid field openings in between targets, each leaf pair does not treat more than one target during one arc. For treatment efficiency, it is attempted to treat as many targets as possible during every arc. The weights of the resulting dynamic conformal arcs are optimized in order to attain the prescribed dose for every lesion with the highest conformity possible. For optimization, an adaptive dose grid with a resolution down to 0.5 mm is used. Final dose distributions are calculated on a 1 mm grid using a pencil beam algorithm. Figure 1 shows the arc setup and a multi-leaf collimator field opening for a sample patient with 6 treated lesions.

### VMAT planning

All patients were re-planned using non-coplanar single isocenter VMAT in the treatment planning system Monaco® (version 5.11., Elekta, Stockholm, Sweden). As for DCAT, the isocenter was set to the geometrical average of the centers-of-mass of all target volumes. Dose distributions were calculated on a 2 mm dose grid using a Monte Carlo algorithm. The same set of couch angles was used for all patients (270°, 300°, 330°, 0°, 30°, 60°). The arc lengths were 360° for the coplanar arc, 180° for the arc at couch angle 270° and 120° for all other arcs. Collimator angles for each arc were chosen on an individual basis by the planner. It was hereby attempted to reduce field configurations with targets aligned along the direction of leaf travel, to avoid multi leaf collimator (MLC) openings in between targets as much as possible. For the optimization, help contours were created by expanding each target by 2 cm. Within these help contours, the Monaco cost functions ‘Conformality’ and ‘Quadratic Overdose’ (with a dose threshold of 10 Gy) were used to maximize conformity and steepness of the dose gradients around the targets as much as possible while maintaining the coverage of 98% of the target volume with the 80% isodose. In the VMAT plans the same OAR dose constraints were used as in the clinically applied single isocenter DCAT plans. Dose constraints were fulfilled for all plans. All plans were reviewed by a senior radiation oncologist and approved as clinically acceptable.

### Plan evaluation

Dose distributions and structures were exported from MBSRS and Monaco®. To provide consistency between the evaluation of the two techniques, both datasets were evaluated using the same in-house tool, which is implemented in MATLAB R2017a (MathWorks Inc., Natick, Massachusetts, USA). It reports dose-volume histogram (DVH) statistics for the defined targets and other regions of interest (ROIs) and uses an open source implementation [15] of the density-based clustering algorithm DBSCAN [16] to identify dose clusters around each lesion. Numbers of monitor units (MUs) as well as the partial volumes of the brain receiving more than 4, 5 and 8 Gy (V_4Gy_, V_5Gy_ and V_8Gy_) were extracted for every plan (including the PTVs). To investigate higher dose levels and metrics such as gradient and conformity indices on a lesion-wise basis, dose clouds of 10 Gy, 12 Gy (V_10Gy_ and V_12Gy_) and half the prescribed dose were identified and assigned to the corresponding lesion. The volumes of those dose clouds were reported excluding the target structures. If lesions were so close to each other that the 10 Gy dose clusters overlapped for one of the techniques, those lesions were joined and treated as one in the subsequent analysis, where V_10Gy_ and V_12Gy_ were compared on a structure-wise basis. Since a gradient index is not defined for neighboring lesions overlapping in their half prescribed isodose, those were excluded from further analysis of target indices, except if they were so close to each other that they also overlapped in their prescribed isodose. In this case, target indices were calculated for the joint structure. For the remaining structures, Paddick conformity index (PCI) and PTV gradient index (GIPTV) were computed and compared. The GIPTV is defined as a tailored version of the Paddick gradient index [17].

$$ GIPTV=\frac{V_{\frac{PI}{2}}}{V_{PTV}} $$

Where ${V}_{\frac{PI}{2}}$ denotes the volume covered by half of the prescribed isodose and *V*_*PTV*_ denotes the volume of the target. This index approaches 1 for an ideal case with an infinitely steep dose fall-off around the target, and values > 1 for realistic dose distributions. The PCI is calculated with the formula [18]:

$$ PCI=\frac{V_{PI, PTV}}{V_{PTV}}\cdot \frac{V_{PI, PTV}}{V_{PI}} $$

Where *V*_*PI*, *PTV*_ denotes the subvolume of the target covered with the prescribed isodose and *V*_*PI*_ denotes the total volume covered with the prescribed isodose, including also regions outside the target. This index takes a value of 1 for perfect conformity and values < 1 for less conformal cases.

To assess a possible predictor of VMAT being superior with respect to healthy brain sparing, the correlation of the sphericity of a lesion with the difference in V_10Gy_ and V_12Gy_ between the two techniques was also assessed for the remaining 53 structures. The sphericity can be used as a measure of how closely a solid resembles a perfect sphere. It is defined as the ratio of the surface of a perfect sphere having the same volume as the solid to the surface of the solid. By expressing the surface *A*_*sphere*_ of the corresponding sphere in terms of the solid’s volume it can be calculated as

$$ \Psi =\frac{A_{sphere}}{A_{solid}}=\frac{\pi^{\frac{1}{3}}{\left(6{V}_{solid}\right)}^{\frac{2}{3}}}{A_{solid}} $$

Where *V*_*solid*_ denotes the volume and *A*_*solid*_ the surface of the solid. Those values were calculated using the built-in functions *alphaShape(), volume()* and *surfaceArea()* in Matlab R2017a.

### Statistics

On a plan-wise basis, the brain volumes receiving more than 4, 5, and 8 Gy and the number of MUs were compared using the Wilcoxon signed rank test (WSRT). On a lesion-wise basis, V_10Gy_ and V_12Gy_ and target indices were compared using WSRT. The correlation of target sphericity and V_10Gy_ / V_12Gy_ was investigated using Spearman’s correlation coefficient. All statistical tests were performed using built-in implementations in Matlab R2017a.

## Results

**Fig. 2:**
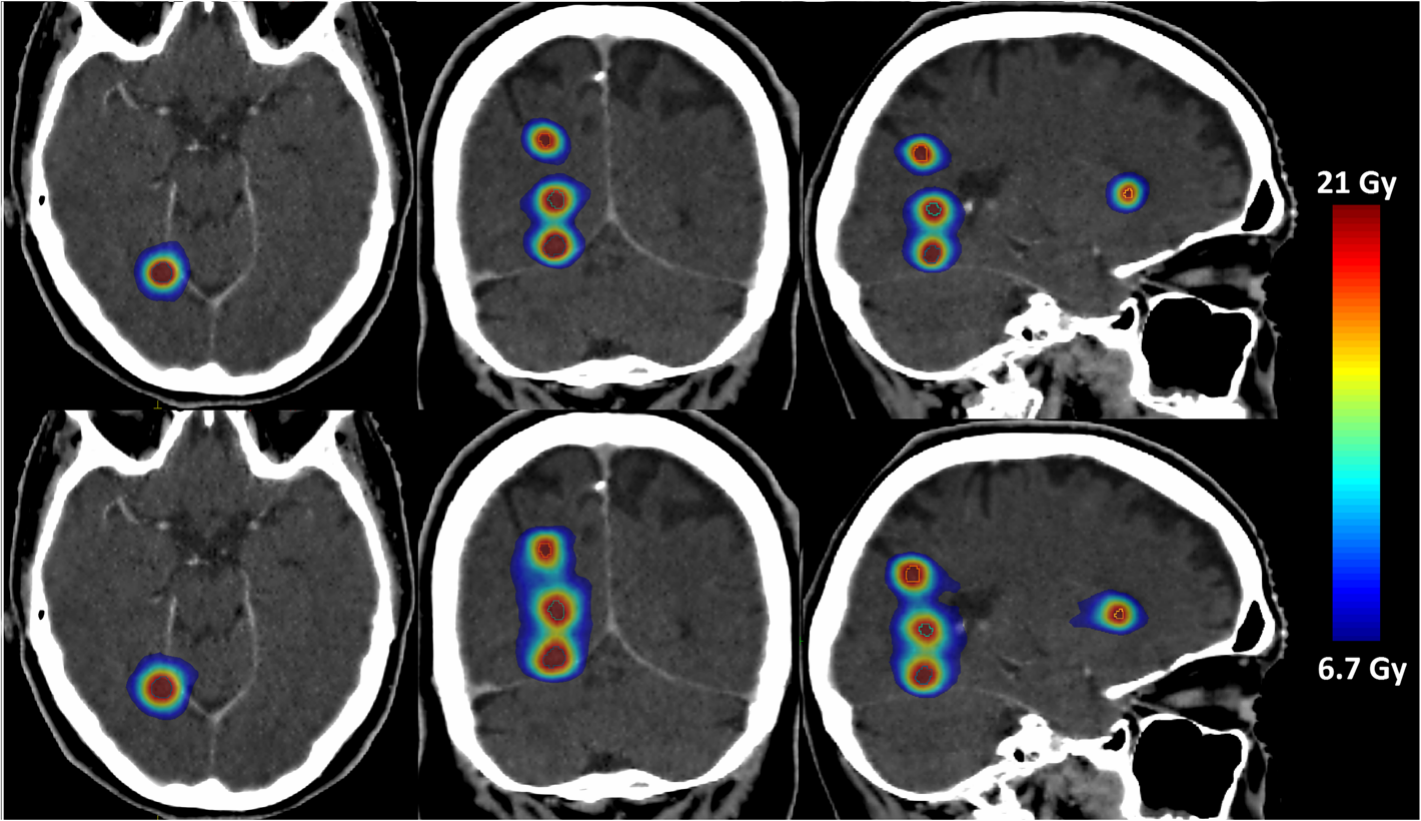
Dose distributions for a patient with 5 metastases (only 4 of them are visible here). The prescribed isodose was 20 Gy for all lesions. The upper three panels show transverse, coronal and sagittal (left to right) slices for the DCAT plan, the lower panels show the respective slices for the VMAT plan. Color wash scales from 6.7 to 21 Gy

**Fig. 3:**
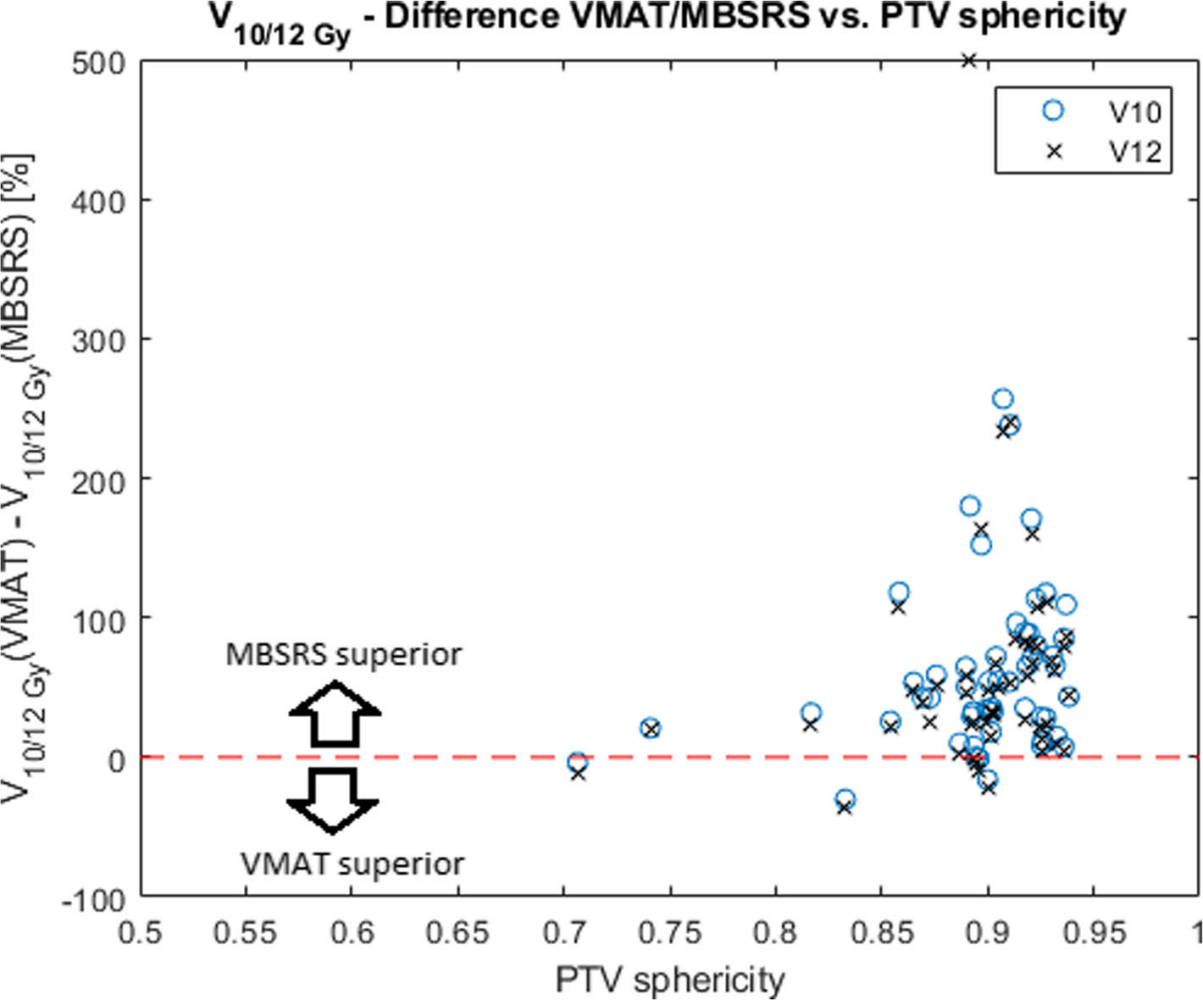
The relative difference in V_10Gy_ and V_12Gy_ between DCAT and VMAT scattered against the sphericity of the corresponding PTV. The reported volumes are normalized to the value obtained with the MBSRS tool. A sphericity value of 1 corresponds to a perfect sphere, values < 1 to shapes deviating from a sphere. Markers above the red dotted line correspond to lesions where DCAT was superior, markers below to lesions where VMAT was superior

Twenty patients with 66 lesions in total who were treated with MBSRS at our institution from January to August 2018 were included in this dosimetric study. Patient characteristics are shown in Table 1 and results are shown in Table 2***.*** For the data evaluated on the plan-wise level (*N* = 20), the V_4Gy_, V_5Gy_ and V_8Gy_ of the brain as well as cumulative MU, were superior for all investigated metrics in the MBSRS plans (*p* < 0.05). In the analysis of the V_10Gy_ and V_12Gy_ for the 66 treated lesions, it occurred 5 times that 2 neighboring lesions overlapped in their corresponding 10 Gy clusters and were evaluated together. There was only one case with 3 neighboring lesions overlapping in their 10 Gy clusters. Therefore, after joining overlapping lesions, the number of investigated lesions was 59. For those 59 targets, volumes of the healthy brain receiving more than 10 and 12 Gy were compared. For this data, MBSRS was superior to VMAT for both V_10Gy_ and V_12Gy_ (*p* < 0.05). For the analysis of target indices, 6 lesions were excluded, because gradient indices could not be calculated. Therefore, 53 targets remained, for which target indices were calculated. Out of these, three were joint structures of two directly neighboring lesions. MBSRS plans had a lower median gradient index of 5.99 compared to the median gradient index of 7.17 for the VMAT plans (*p* < 0.05). The differences in the conformity index were minimal (median 0.75 vs. 0.73 for MBSRS and VMAT, respectively) and only slightly below the significance threshold (*p* = 0.04). A comparison of the dose distribution for an exemplary case with 6 lesions is shown in Fig. 2, where the steeper dose gradient for MBSRS results in a visible separation of the lesions in the color wash visualization. A moderate correlation between the sphericity and the difference in the V_10Gy_ and V_12Gy_ was found (Spearman’s *ρ* = 0.27 and 0.30, for V_10Gy_ and V_12Gy_, respectively and *p* < 0.05 for both parameters). A scatterplot is shown in Fig. 3*.* Target structures with a lower sphericity tended to show a smaller advantage of MBSRS compared to VMAT, or even a superiority of VMAT, with respect to V_10Gy_ and V_12Gy_.

**Table 1:** Patient characteristics

Number of patients	20
Sex	Male: 10, Female: 10
Median age at RT [years]	62, range: 51–77
Total number of treated lesions	66
Median number of treated lesions per patient	3, range: 2–6
Median target volume [cm^3^]	0.8, range: 0.1–11.9
Median prescribed isodose [Gy]	19, range: 15–20

**Table 2:** Results for all investigated metrics. While some metrics were investigated on a plan-wise basis (cumulative MU, and the brain receiving 4, 5 and 8 Gy) others were examined on a lesion-wise basis (V_10Gy_, V_12Gy_, conformity index, gradient index)

	MBSRS DCAT median (range)		Monaco® VMAT median (range)		*P* (Wilcoxon SR)
Plan data *N* = 20		*# plans MBSRS superior*		*# plans VMAT superior*	
Cumulative MU	4569 (3543–7790)	17	5840 (4089–10,769)	3	< 0.05
V_4Gy_ (brain) [cm^3^]	45.6 (18.5–215.9)	19	123.1 (37.5–418.6)	1	< 0.05
V_5Gy_ (brain) [cm^3^]	33.7 (13.0–120.5)	19	69.1 (26.0–273.6)	1	< 0.05
V_8Gy_ (brain) [cm^3^]	17.4 (6.3–52.6)	19	26.6 (10.6–86.4)	1	< 0.05
PTV data *N* = 59		*# PTVs MBSRS superior*		*# PTVs VMAT superior*	
V_10Gy_ (healthy brain) [cm^3^]	3.2 (0.4–19.3)	55	4.9 (1.0–19.9)	4	< 0.05
V_12Gy_ (healthy brain) [cm^3^]	2.1 (0.1–13.1)	53	3.1 (0.5–13.9)	6	< 0.05
PTV data without PTVs overlapping in half prescribed isodose *N* = 53		*# PTVs MBSRS superior*		*# PTVs VMAT superior*	
Paddick CI	0.75 (0.58–0.89)	30	0.73 (0.38–0.88)	22	< 0.05
GIPTV	5.99 (3.50–15.73)	48	7.17 (3.35–33.00)	5	< 0.05

## Discussion

The purpose of this study was to compare two single isocenter techniques for single fraction SRS for multiple brain metastases: Monaco® VMAT and DCAT by means of MBSRS. A significant reduction of MUs, the volume of the brain receiving more than 4, 5 and 8 Gy, the volumes of healthy brain tissue around each lesion receiving more than 10 and 12 Gy and the gradient index were observed for MBSRS. Possibly, the decreased low dose spread for MBSRS can be explained by the pre-selection of targets to be treated through each arc described above, which minimizes MLC openings in between targets. In VMAT, there is no such restriction and any target can be treated by any of the arcs. Conformity was comparable for both techniques. Since, contrarily to VMAT, MBSRS does not treat all targets through each arc, a higher number of arcs is necessary for MBSRS (in our study 10–12 while for VMAT only 6 arcs were used). However, due to the high modulation in the VMAT plans, the overall number of MUs was still significantly lower for MBSRS. To the best of our knowledge, this study is the first to compare MBSRS to VMAT on Elekta LINACs. Narayanasamy et al. conducted a similar study comparing MBSRS to RapidArc™ on a Varian LINAC for 8 patients [19]. Their results are in agreement with our study with respect to the number of MUs and gradient indices; however, they did not report a substantial reduction of the V_12Gy_ for MBSRS. Gevaert et al. also compared MBSRS to RapidArc™ and to conventional multi isocenter DCAT in a dosimetric study with 10 patients [20]. In agreement with our results, the authors reported a lower V_10Gy_ and V_12Gy_ for both MBSRS and conventional DCAT compared to RapidArc™, as well as a lower gradient index. We also discovered a moderate correlation of the target sphericity to the difference in V_10Gy_ and V_12Gy_ between the two techniques. Recently, also a study comparing MBSRS to Varian HyperArc™ (HA), a dedicated VMAT technique for treatment of multiple brain metastases has been published by Ruggieri et al. [21]. An equivalence between the two techniques was reported for most investigated metrics, except for the conformity, which was superior with HA. In our study, for lesions of lower sphericity, there is a tendency towards less advantage of MBSRS or even a superiority of VMAT. Since in our patient cohort most lesions were small (median target volume: 0.8 cm^3^) and of high sphericity, further evaluation of cases with larger and more irregularly shaped lesions is warranted to support this finding. Furthermore, it was a retrospective study with patients selected for treatment with DCAT without control group. Prospective data is needed to confirm our results.

The most severe adverse effect after cerebral radiation therapy is radiation necrosis [22]. Especially the application of high prescription doses in one single fraction, as used in SRS, has a high risk of radiation necrosis of up to 10% [23]. V_10Gy_ and V_12Gy_ are one of the most important parameters to estimate the risk for radiation necrosis [24–26]. Therefore, both techniques were also analyzed with respect to these two values. Important hereby are the coherent areas of V_10Gy_ and V_12Gy,_ why adjacent lesions with overlapping 10 Gy clusters were evaluated together. According to our results, the use of DCAT through MBSRS could provide a lower risk for radiation necrosis than VMAT, especially if the metastases are nearly spherical. However, this study only provides dosimetric results. In order to show a reduced risk of radiation necrosis when using DCAT on multiple metastases, further prospective data is needed. Given that the DCAT plans also have advantages in terms of plan complexity and overall number of MUs and therefore treatment time, the technique could be a valuable option for SRS treatment of multiple brain metastases.

## Conclusions

In single isocenter SRS for multiple brain metastases, MBSRS can often generate treatment plans with steeper dose gradients, superior healthy brain sparing and less MUs as compared to VMAT plans, in particular if the lesions are nearly spherical. This could result in a possible reduction of the risk of radiation necrosis. VMAT can be superior in selected cases with irregularly shaped target volumes, where the shape of lesions deviates substantially from a sphere.

## Data Availability

The datasets supporting the conclusions of this article are included within the article.

## Abbreviations

CT: Computed tomography
DCAT: Dynamic conformal arc therapy
DVH: Dose-volume histogram
GIPTV: PTV gradient index
HA: HyperArc™
LINAC: Linear accelerator
MBSRS: Multiple Brain Mets SRS®
MLC: Multi leaf collimator
MPRAGE: Magnetization-prepared rapid gradient-echo
MRI: Magnetic resonance imaging
MU: Monitor unit
OAR: Organ-at-risk
PCI: Paddick conformity index
PTV: Planning target volume
ROI: Region of interest
SRS: Stereotactic radiosurgery
VMAT: Volumetric modulated arc therapy
WBRT: Whole brain radiotherapy
WSRT: Wilcoxon signed-rank test
